# Effect of Stir-Frying, Boiling, and Baking on Hexaconazole Residue Levels in Welsh Onion (*Allium fistulosum* L.)

**DOI:** 10.3390/foods14020168

**Published:** 2025-01-08

**Authors:** Myungheon Kim, Mihyun Cho, Jaebin Im, Changkyo Seo, Changhyeon Park, Moo-Hyeog Im

**Affiliations:** Department of Food Engineering, Daegu University, Gyeongsan 38453, Republic of Korea

**Keywords:** hexaconazole, Welsh onion, residue, stir-frying, boiling, baking

## Abstract

Hexaconazole, a triazole insecticide, is widely used to control rust disease in Welsh onions. Residue levels of pesticides vary based on the cooking methods. Although studies on hexaconazole residue have involved vegetables such as cabbage, research on Welsh onion is limited. This study investigated the effect of different cooking methods on hexaconazole residues. Welsh onion was processed by common cooking methods such as stir-frying, boiling, and baking, and analyzed for pesticide residues using liquid chromatography with tandem mass spectrometry. The results showed that the removal rates of pesticides after cooking were 40.0–62.9% for stir-frying, 80.0–91.4% for boiling, and 51.4–77.1% for baking. Regardless of cutting thickness (0.8 cm or 5 cm), the reduction rate of pesticide residues increased with time during boiling. Increased reduction rates were also observed with increased time during baking. As stir-frying time increased, the residue amount increased due to water loss. However, the absolute amount of the pesticide decreased with increasing time. Therefore, the residue amount of hexaconazole in Welsh onion was reduced by various general cooking processes. These findings can provide a valuable foundation for research on Welsh onion processing, address consumer concerns about pesticide residues, and provide baseline data for risk assessments.

## 1. Introduction

In agriculture, pesticides are essential for controlling pests, diseases, or weeds and serve as important agricultural inputs for increasing yield, improving quality, and reducing labor [1]. However, the misuse of pesticides can lead to residues exceeding permitted levels, resulting in their accumulation in the body and potentially causing chronic toxicity. Along with environmental pollution, these issues lead to a negative consumer perception of pesticides, a trend that has been increasing recently.

In 2015, the International Agency for Research on Cancer (IARC) classified glyphosate as “probably carcinogenic to humans”; this amplified public fears [2] and increased consumer anxiety, leading to the emergence of a new term, “chemiphobia” [3]. The amount of pesticide residues in agricultural products is very little, and considering the daily intake of these products, the possibility of causing acute toxicity is extremely low. Nevertheless, since foods, including agricultural products, are consumed over a lifetime, the chronic toxicity of pesticides may become a problem as the detection frequency and level increases [4]. Based on national nutrition statistics, vegetables have the highest average intake in Korea (272 g), followed by grains and their products (261 g), and beverages (207 g). This indicates that vegetable consumption surpasses the consumption of grains, which are traditionally considered staple foods [5]. Additionally, a safety survey conducted by the National Agricultural Products Quality Management Service reported that vegetables have the highest non-compliance rate at 2.2%. Since most vegetables have a short growing period and a large amount of pesticides adhere to the crop surface, there is a high possibility that pesticides will remain in leafy vegetables [6]. Vegetables are produced through intensive cultivation methods to maximize yield per unit area, and since consumers often consume them raw, they are at a higher risk of direct exposure to harmful chemicals such as pesticides [7]. Therefore, to protect consumers from pesticide residues in vegetables, a comprehensive assessment of pesticides, including characteristics, concentrations, and consumption patterns, is necessary to evaluate exposure levels and the risks associated with vegetable consumption.

In Korea, Good Agricultural Practices have been implemented to ensure the safety of agricultural products to enhance consumer trust, strengthen international competitiveness, and support a sustainable agricultural environment [8]. To ensure the safe use of pesticides in crop cultivation, pesticide safety standards have been established for each crop, along with regulated maximum residue limits (MRLs) at levels deemed safe for daily lifelong consumption by the public without adverse health effects [3]. Currently, the Ministry of Food and Drug Safety sets pesticide MRLs in foods by ensuring that the proposed limits do not exceed the acceptable daily intake for consumers, based on pesticide residue data from raw agricultural products. The standards for theoretical maximum daily intake are calculated using the national average food intake levels [3,9].

Welsh onion (*Allium fistulosum* L.) is a perennial herbaceous plant in the Liliaceae family, known for its strong cold and heat tolerance, with an optimal growth temperature of 15–25 °C, making it well suited for monoculture in cool climates. It is also one of the primary seasoning vegetables cultivated in Korea. Traditionally, Welsh onions are harvested and sold in bundles directly from the field; however, recently, the trend has shifted toward packaging after initial trimming, leading to an increase in individually packaged products in the market [10,11]. In addition, Welsh onion is an important vegetable in regions such as Northeast Asia, including Korea, Europe, and North America [12].

Currently, 142 types of pesticides are registered for use on Welsh onions in Korea [13]. Among these is hexaconazole, a triazole insecticide widely used globally, developed by Zeneca in the United Kingdom, and a systemic pesticide effective for both preventive and curative control of ascomycetes and basidiomycetes. In Korea, hexaconazole is used to control sheath blight, powdery mildew, and rust. It has the following physicochemical properties: a molecular weight of 314.2 g/mol, an octanol–water partition coefficient (log Kow) of 3.9, a water solubility of 0.17 g/L, a vapor pressure of 0.018 mPa at 20 °C, and a residue limit of 0.1 mg/kg for Welsh onion [14,15].

Pesticide residue analyses are conducted to ensure the supply of safe agricultural products to consumers. Since most agricultural products are consumed after cooking or processing, it is essential to consider changes in residue levels throughout the cooking process [16]. Kim et al. [3] reported that in tests on changes in azoxystrobin residue levels during the processing of eggplant and lettuce, nearly 60% of the pesticide residue was removed through washing alone. Furthermore, the study by Ryu et al. (2017) [15] demonstrated that during the blanching process of cabbage, hexaconazole residues were reduced by 79.5%, with blanching resulting in a greater reduction in residue levels compared to those with washing (67.6%) and salting (68.9–74.8%). While studies on the residue analysis and persistence of hexaconazole [17], behaviors in soil and crops [18], and metabolism [19] have been conducted, research on the relationship between hexaconazole and Welsh onion processing remains limited.

Therefore, this study aimed to examine the residue behavior of hexaconazole in Welsh onions during common household cooking methods like stir-frying, boiling, and baking to provide foundational data for future research on Welsh onion processing.

## 2. Materials and Methods

### 2.1. Sample and Reagents

Welsh onions grown in Gyeongsan-si in 2022 were purchased from the market in Hayang-eup, Gyeongsan-si, for this study. The purchased Welsh onions were confirmed as controls, with no detectable hexaconazole residues, using the analytical method employed in this study. Hexaconazole (Kemidas, Gunpo, Republic of Korea) was purchased as a standard substance at a concentration of 1000 μg/mL. Acetonitrile for HPLC analysis (J. T. Baker, Radnor, PA, USA), magnesium sulfate (MgSO_4_, 99.5%), and sodium acetate (NaOAc, 99.5%) were obtained from Sigma-Aldrich (Maryland Heights, MI, USA). Primary secondary amine (PSA) was purchased from Agilent Technologies (Santa Clara, CA, USA). For instrumental analysis, ammonium acetate and formic acid (≥98%) from Sigma-Aldrich (Maryland Heights, MI, USA) were used as mobile phase solvents, while water and methanol were sourced from J. T. Baker.

### 2.2. Sample Preparation

After purchasing Welsh onions from the market, the absence of detectable pesticide residues was confirmed before use. In the process of cooking Welsh onion, the outer skin is typically removed before preparation. Accordingly, in this study, the outer skin was removed, and the Welsh onion was immersed in a pesticide solution to simulate residue retention, aligning with actual processing methods. Forty liters of immersion solution was prepared with 10 mg/L hexaconazole pesticide (2% SC, Samgong Hexaconazole, Hankooksamgong, Seoul, Republic of Korea). Immersion was used to observe changes in residue levels because actual cultivated Welsh onions often exhibit significant variations in pesticide residues. Welsh onions were soaked in the solution for 10 s and then air-dried at room temperature for 30 min before they were subjected to cooking processes. Similarly to this study, many research papers investigating changes in pesticide residues in agricultural products have adopted the immersion method.

### 2.3. Cooking of Welsh Onions

Before the cooking processes (stir-frying, boiling, and baking), five Welsh onions were washed under running water for 1 min at a flow rate of 6 L/min. After completing the cooking processes, the samples were ground using a blender (Grinmic Gold-DA10000G, Daesung Artlon, Paju, Republic of Korea) and stored in a freezer at −20 °C until pesticide residue analysis.

#### 2.3.1. Stir-Frying

Welsh onions were cut into 8 mm slices, and 400 g samples were taken. After adding 8 mL of refined vegetable oil used for cooking, the sample was placed in a preheated pan at 180 °C and stir-fried for 30 s and 1, 3, and 5 min.

#### 2.3.2. Boiling

Welsh onions were cut into lengths of 8 mm or 5 cm, and 400 g of each was placed into 10 L of boiling water. The 8 mm slices were boiled for 1, 3, 5, 7, and 10 min, while the 5 cm slices were boiled for 5, 10, 15, and 20 min.

#### 2.3.3. Baking

Welsh onions were cut into 5 cm lengths and placed in an oven (Phantom Electric Deck Oven, Sam Jung Enterprise Co., Hanam, Republic of Korea) preheated to 180 °C and baked for 5, 10, 15, and 20 min.

### 2.4. Pesticide Residue Analysis

A 10 g homogenized Welsh onion sample was placed into a 50 mL conical tube, followed by the addition of 10 mL of acetonitrile containing 1% acetic acid. The mixture was shaken for 3 min at 2000 rpm. The sample was shaken for 1 min after adding 6 g of MgSO_4_ and 1.5 g of NaOAc, and then centrifuged for 10 min at 4000× *g*. Then, 150 mg of MgSO_4_ and 50 mg of PSA were added into 1 mL of the supernatant and centrifuged after 30 s of shaking. The filtered supernatant (0.2 μm PTFE membrane filter, Agela Technologies, Tianjin, China) was analyzed by liquid chromatography with tandem mass spectrometry (LC-MS/MS, Agilent 6470 Triple Quad LC/MS, Agilent Technologies, Santa Clara, CA, USA) for hexaconazole. The Agilent Eclipse Plus C18 column (100 mm × 2.1 mm × 1.8 μm) was used, and samples were transported using mobile phases consisting of water (containing 5 mM ammonium acetate and 0.1% formic acid) and methanol.

### 2.5. Conditions for Instrumental Analysis

Pesticide residues in Welsh onions were analyzed using LC–MS/MS. LC was performed using a C18 column (100 mm × 2.1 mm, 1.8 μm, Agilent Eclipse Plus) (Santa Clara, CA, USA). Two mobile phases, A (water, 0.1% formic acid, and 5 mM ammonium acetate) and B (methanol, 0.1% formic acid, and 5 mM ammonium acetate), were used at a flow rate of 0.2 mL/min. The gradient was started at 10% B for 1 min and increased to 55% B over 0.5 min, 60% B over the next 3.5 min, and 98% B within the following 5 min; it was maintained at 98% B for 5 min. Finally, it was returned to 10% B over 0.1 min and maintained for 4.9 min. The total duration of the procedure was 20 min. MS/MS was conducted in the positive electron spray ionization mode with an interface voltage of 3.5 kV. The multiple reaction monitoring conditions for the compound were as follows: precursor ion (*m*/*z*), 314.1 and product ion (*m*/*z*), 158.9 and 70.1. The corresponding collision energy values were 36 and 24, respectively.

### 2.6. Method Validation

To verify the accuracy and reproducibility of the hexaconazole analysis method, a recovery test was conducted on Welsh onions in which pesticide residues were not detected. For the recovery test, 10 g of homogenized samples was mixed with hexaconazole standard solution at the limit of quantification (LOQ) and at 10 and 50 times the LOQ. The recovery rates were analyzed in triplicate and analytical errors were then evaluated. Linearity was assessed by constructing matrix-matched calibration curves and calculating the coefficient of determination (R^2^). The limit of detection (LOD) and LOQ were determined by analyzing control samples and calculating the signal-to-noise (S/N) ratio of chromatographic peaks. Peaks with an S/N ratio of >3 were defined as the LOD, whereas those with an S/N ratio of >10 were defined as the LOQ.

### 2.7. Statistical Analysis

All experiments in this study were conducted in triplicate, and results were expressed as mean ± standard deviation. Statistical analysis was performed using the SAS program (ver. 9.4, SAS Institute Inc., Cary, NC, USA). The significance among samples was tested by one-way analysis of variance and Duncan’s multiple range test was applied at a significance level of *p* < 0.05.

## 3. Results and Discussion

### 3.1. Validation of the Analytical Method

To analyze hexaconazole residues in Welsh onions, we evaluated the coefficient of determination (R^2^) of the calibration curve, as well as the recovery rate and relative standard deviation (RSD) of the method. The R^2^ of the matrix-matched calibration curve was excellent, exceeding 0.99. The LOD and LOQ for hexaconazole were 0.003 mg/kg and 0.01 mg/kg, respectively. Hexaconazole standard solution was spiked into non-detected samples at concentrations of LOQ (0.01 mg/kg), 10 × LOQ (0.1 mg/kg), and 50 × LOQ (0.5 mg/kg), and each sample was analyzed in triplicate, with results shown in Table 1. The mean recovery rates at the LOQ level were 94.1–102.6% for raw, stir-fried, boiled, and baked samples; 90.4–102.9% at the 10 × LOQ level; and 89.4–108.8% at the 50 × LOQ level. The RSDs were all confirmed to be below 3.0%, meeting the residue analysis standards specified in the “Guidelines on Standard Procedures for Food Testing Methods (2016)” by the Ministry of Food and Drug Safety [20] and the CODEX guidelines (CAC/GL 40-1993) [21].

### 3.2. Residue Behavior of Pesticide During Stir-Frying

The residue levels and reduction rates of hexaconazole in Welsh onions, depending on stir-frying time, are presented in Table 2. The initial residue level of hexaconazole in Welsh onions was 0.35 mg/kg, which decreased to 0.19 mg/kg after washing under running water for 1 min just before processing. When stir-frying times were set at 30 s, 1 min, 3 min, and 5 min, the residue levels were 0.13, 0.13, 0.18, and 0.21 mg/kg, respectively, with reduction rates of 62.9%, 62.9%, 48.6%, and 40.0%, respectively. The moisture content at each time point was 89.3%, 89.2%, 84.6%, and 81.2%, and the absolute pesticide amounts considering moisture content were 1.25, 1.24, 1.19, and 1.12 mg/kg, respectively. There was no significant difference in hexaconazole residue levels and reduction rates between 30 s and 1 min (*p* < 0.05). Therefore, the increase in residue levels and decreased reduction rate from prolonged stir-frying resulted from the moisture loss of Welsh onions. According to Zhao et al. [22], the residue levels of 13 pesticides decreased in lettuce, tomatoes, and cucumbers after 5 min of stir-frying, although some compounds became concentrated. This was attributed to prolonged heating with a small amount of oil, leading to moisture evaporation and concentration of certain pesticides. Kim et al. [3] reported a 75.7% reduction in azoxystrobin, a systemic pesticide, when applied to eggplants and stir-fried for 1 min. In contrast, Radwan et al. [23] observed a reduction of over 98.5% of profenofos in peppers, paprika, and eggplant after 5 min of stir-frying. Profenofos, a non-systemic pesticide with a log Kow of 4.44 and a vapor pressure (VP) of 2.53 (at 20 °C), exhibited higher lipophilicity than hexaconazole but also greater volatility, likely contributing to significant pesticide removal during stir-frying as it does not penetrate the plant tissues. Based on previous findings, it can be concluded that when systemic pesticides are applied to agricultural products, shorter stir-frying times result in greater pesticide reduction. However, with longer cooking times, moisture evaporation can lead to a concentration effect, although the absolute pesticide amount ultimately decreases.

### 3.3. Residue Behavior of Pesticide During Boiling

The results of pesticide residue levels and reduction rates according to the size of Welsh onions and boiling time are presented in Table 3. When Welsh onions cut into 8 mm slices were boiled for 1, 3, 5, 7, and 10 min, the residue levels were 0.07, 0.06, 0.03, 0.03, and 0.03 mg/kg, respectively, with removal rates of 82.9, 82.9, 91.4, 91.4, and 91.4%, respectively. The moisture content at each time point was 92.6, 92.1, 93.0, 92.4, and 91.6%, respectively, with corresponding absolute pesticide amounts of 0.86, 0.76, 0.43, 0.40, and 0.37 mg/kg. For 5 cm samples boiled for 5, 10, 15, and 20 min, the residue levels were 0.06, 0.05, 0.03, and 0.03 mg/kg, with reduction rates of 80.0, 85.7, 91.4, and 91.4%, respectively. The moisture content at each time point was 91.6, 91.1, 91.4, and 92.2%, respectively, with corresponding absolute pesticide amounts of 0.83, 0.56, 0.35, and 0.34 mg/kg, respectively. Compared to initial residue levels, the 8 mm samples showed reduction rates of 79.3–91.0%, and the 5 cm samples showed reduction rates of 79.8–91.8%, indicating that pesticide residues decreased during boiling regardless of sample size. According to the studies by Suntio et al. [24] and Kwon et al. [25], pesticides with higher Henry’s law constants are more likely to volatilize into the atmosphere, and pesticide removal during boiling has been reported to correlate with Henry’s law constant. Kwon et al. [25] found that when spinach was boiled for 1, 3, and 5 min, bifenthrin was reduced by 66–76%, metalaxyl by 96–98%, and procymidone by 87–91%, with longer boiling times resulting in greater pesticide reduction. Although the Henry’s law constants for bifenthrin and procymidone are higher at 7.74 × 10^−5^ and 2.65 × 10^−3^ Pa m^3^ mol^−1^, respectively, than those for metalaxyl (1.60 × 10^−5^ Pa m^3^ mol^−1^), metalaxyl showed the highest removal rate. This suggests that in boiling water, pesticides with lower Henry’s law constants tend to transfer more readily into the water as boiling time increases, leading to higher removal rates. In this study, hexaconazole, which has a relatively low Henry’s law constant (3.33 × 10^−4^ Pa m^3^ mol^−1^), also showed increased residue transfer into the water with longer boiling times, consistent with the findings of the above studies, resulting in a higher pesticide removal rate.

### 3.4. Residue Behavior of Pesticide During Baking

The residue levels and removal rates of pesticides for Welsh onions cut into 5 cm slices and baked for 5, 10, 15, and 20 min are presented in Table 4. As baking time increased, the residue levels decreased to 0.17, 0.11, 0.10, and 0.08 mg/kg, with removal rates of 51.4%, 68.6%, 71.4%, and 77.1%, respectively. The moisture content at each time point was 89.2%, 87.6%, 85.7%, and 81.7%, respectively, with corresponding absolute pesticide amounts of 1.55, 0.91, 0.72, and 0.45 mg/kg, respectively. These results show that the removal rate of hexaconazole increased with longer baking times, confirming that the baking process effectively reduces pesticide residues in Welsh onions. In a study by Habiba et al. [26], potatoes treated with profenofos and then oven-baked showed a 98% reduction in residue compared to the initial levels. Although the pesticide used in this study is less volatile and demonstrates systemic properties, it is less lipophilic than profenofos, contributing to the observed residue reduction. Additionally, during baking, the substrate (Welsh onions) is exposed to heat, which can lead to pesticide reduction through evaporation, co-distillation, and thermal decomposition, depending on the pesticide’s characteristics [27]. In this study, the heat from baking likely caused a co-distillation effect, where the water content in Welsh onions evaporated along with the pesticide compounds, reducing hexaconazole residue levels. This effect may increase volatility or enable the pesticide to transfer with water vapor, thereby decreasing hexaconazole residues.

## 4. Conclusions

This study analyzed the residues of hexaconazole in Welsh onions during stir-frying, boiling, and baking using LC-MS/MS. The residue level of Welsh onions before processing was 0.35 mg/kg, which decreased to 0.19 mg/kg after washing. During stir-frying, the removal rate of hexaconazole ranged from 40.0% to 62.9%, with longer cooking times leading to moisture evaporation and the concentration of pesticide residues, though the absolute pesticide amount, adjusted for moisture content, showed a decreasing trend. In boiling, residue levels decreased regardless of sample size, with removal rates of 79.3–91.0% for 8 mm samples and 79.8–91.8% for 5 cm samples depending on boiling time. Baking resulted in removal rates of 62.5–89.0% as cooking time increased. Therefore, the optimal cooking times for effective pesticide removal were 5 min for stir-frying, 5 min for thinly sliced samples and 15 min for thickly sliced samples during boiling, and over 20 min for baking. The future monitoring of Welsh onions during distribution, along with subsequent dietary risk assessments, is expected to enhance consumer confidence in the safety of their consumption.

## Figures and Tables

**Table 1:** Recovery rate of hexaconazole in Welsh onion and its processed products (*n* = 3).

Sample	Fortification (mg/kg)	Recovery (%) ± SD ^1^	RSD ^2^ (%)	LOQ ^3^ (mg/kg)
Raw Welsh onion	0.01	94.9 ± 0.9	1.0	0.01
	0.1	98.1 ± 0.3	0.3	
	0.5	100.4 ± 0.6	0.6	
Stir-frying	0.01	94.1 ± 1.9	2.0	0.01
	0.1	90.4 ± 1.0	1.1	
	0.5	89.4 ± 2.7	3.0	
Boiling	0.01	102.6 ± 2.5	2.4	0.01
	0.1	102.9 ± 1.8	1.7	
	0.5	108.8 ± 1.3	1.2	
Baking	0.01	94.9 ± 1.4	1.4	0.01
	0.1	100.5 ± 0.6	0.6	
	0.5	102.1 ± 0.9	0.8	

^1^ SD: standard deviation. ^2^ RSD: Relative standard deviation. ^3^ LOQ: Limit of quantitation.

**Table 2:** Removal efficiency of hexaconazole residues from Welsh onion by stir-frying.

Cooking Method		Residue Level (mg/kg) ± SD ^1^ (Wet Basis)	Removal Rate ^2^ (%)	Moisture Content (%)	Residue Level (mg/kg) ± SD (Dry Basis)	Removal Rate ^3^ (%)
Raw Welsh onion		0.35 ± 0.01 ^a^	-	91.5	4.13 ± 0.12 ^a^	-
Running water wash for 1 min		0.19 ± 0.01 ^c^	45.7	89.7	1.80 ± 0.06 ^b^	56.3
Stir-frying (min)	0.5	0.13 ± 0.01 ^e^	62.9	89.3	1.25 ± 0.14 ^c^	69.8
	1	0.13 ± 0.01 ^e^	62.9	89.2	1.24 ± 0.13 ^c^	70.1
	3	0.18 ± 0.01 ^d^	48.6	84.6	1.19 ± 0.09 ^c^	71.2
	5	0.21 ± 0.02 ^b^	40.0	81.2	1.12 ± 0.09 ^c^	73.0

^1^ SD: standard deviation. ^2^ Removal rate calculated based on hexaconazole residue in raw Welsh onion (wet basis). ^3^ Removal rate calculated based on hexaconazole residue in raw Welsh onion (dry basis). ^a–e^ Values followed by the same superscript letter indicate no statistically significant difference (*p* < 0.05).

**Table 3:** Removal efficiency of hexaconazole residues from Welsh onion by boiling.

Cooking Method			Residue Level (mg/kg) ± SD ^1^ (Wet Basis)	Removal Rate ^2^ (%)	Moisture Content (%)	Residue Level (mg/kg) ± SD (Dry Basis)	Removal Rate ^3^ (%)
Raw Welsh onion			0.35 ± 0.01 ^a^	-	91.5	4.13 ± 0.12 ^a^	-
Running water wash for 1 min			0.19 ± 0.01 ^b^	45.7	89.7	1.80 ± 0.06 ^b^	56.3
Boiling (min)	8 mm	1	0.07 ± 0.00 ^c^	82.9	92.6	0.86 ± 0.08 ^c^	79.3
		3	0.06 ± 0.00 ^d^	82.9	92.1	0.76 ± 0.00 ^d^	81.5
		5	0.03 ± 0.00 ^f^	91.4	93.0	0.43 ± 0.00 ^e^	89.7
		7	0.03 ± 0.00 ^f^	91.4	92.4	0.40 ± 0.00 ^f^	90.4
		10	0.03 ± 0.00 ^f^	91.4	92.0	0.37 ± 0.00 ^g^	91.0
	5 cm	5	0.06 ± 0.01 ^d^	80.0	91.6	0.83 ± 0.00 ^c^	79.8
		10	0.05 ± 0.00 ^e^	85.7	91.1	0.56 ± 0.00 ^d^	86.5
		15	0.03 ± 0.00 ^f^	91.4	91.4	0.35 ± 0.00 ^e^	91.6
		20	0.03 ± 0.00 ^f^	91.4	92.2	0.34 ± 0.00 ^f^	91.8

^1^ SD: standard deviation. ^2^ Removal rate calculated on the basis of hexaconazole residue in raw Welsh onion (wet basis). ^3^ Removal rate calculated on the basis of hexaconazole residue in raw Welsh onion (dry basis). ^a–g^ Values followed by the same superscript letter indicate no statistically significant difference (*p* < 0.05).

**Table 4:** Removal efficiency of hexaconazole residues from Welsh onion by baking.

Cooking Method		Residue Level (mg/kg) ± SD ^1^ (Wet Basis)	Removal Rate ^2^ (%)	Moisture Content (%)	Residue Level (mg/kg) ± SD (Dry Basis)	Removal Rate ^3^ (%)
Raw Welsh onion		0.35 ± 0.01 ^a^	-	91.5	4.13 ± 0.12 ^a^	-
Running water wash for 1 min		0.19 ± 0.01 ^b^	45.7	89.7	1.80 ± 0.06 ^b^	56.3
Baking (min)	5	0.17 ± 0.02 ^c^	51.4	89.2	1.55 ± 0.21 ^c^	62.5
	10	0.11 ± 0.01 ^d^	68.6	87.6	0.91 ± 0.05 ^d^	77.9
	15	0.10 ± 0.01 ^d^	71.4	85.7	0.72 ± 0.04 ^e^	82.5
	20	0.08 ± 0.01 ^e^	77.1	81.7	0.45 ± 0.03 ^f^	89.0

^1^ SD: standard deviation. ^2^ Removal rate calculated on the basis of the hexaconazole residue in raw Welsh onion (wet basis). ^3^ Removal rate calculated on the basis of the hexaconazole residue in raw Welsh onion (dry basis). ^a–f^ Values followed by the same superscript letter indicate no statistically significant difference (*p* < 0.05).

## Data Availability

The original contributions presented in the study are included in the article, and further inquiries can be directed to the corresponding author.
